# Mitochondrial oxidative stress caused by Sod2 deficiency promotes cellular senescence and aging phenotypes in the skin

**DOI:** 10.18632/aging.100423

**Published:** 2012-01-20

**Authors:** Michael C. Velarde, James M. Flynn, Nicholas U. Day, Simon Melov, Judith Campisi

**Affiliations:** ^1^ Buck Institute for Research on Aging, Novato, CA 94945, USA; ^2^ Lawrence Berkley National Laboratory, Berkeley, CA 94720, USA

**Keywords:** DNA damage, epidermal differentiation, knock-out mouse model, reactive oxygen species (ROS), superoxide

## Abstract

Cellular senescence arrests the proliferation of mammalian cells at risk for neoplastic transformation, and is also associated with aging. However, the factors that cause cellular senescence during aging are unclear. Excessive reactive oxygen species (ROS) have been shown to cause cellular senescence in culture, and accumulated molecular damage due to mitochondrial ROS has long been thought to drive aging phenotypes *in vivo*. Here, we test the hypothesis that mitochondrial oxidative stress can promote cellular senescence *in vivo* and contribute to aging phenotypes *in vivo*, specifically in the skin. We show that the number of senescent cells, as well as impaired mitochondrial (complex II) activity increase in naturally aged mouse skin. Using a mouse model of genetic *Sod2* deficiency, we show that failure to express this important mitochondrial anti-oxidant enzyme also impairs mitochondrial complex II activity, causes nuclear DNA damage, and induces cellular senescence but not apoptosis in the epidermis. *Sod2* deficiency also reduced the number of cells and thickness of the epidermis, while increasing terminal differentiation. Our results support the idea that mitochondrial oxidative stress and cellular senescence contribute to aging skin phenotypes *in vivo*.

## INTRODUCTION

Cellular senescence is an important anti-cancer mechanism that arrests the proliferation of cells in the face of potentially oncogenic stress [1]. Cellular senescence has also been implicated in mammalian aging and age-related diseases in numerous tissues [2-5]. In mice and humans, cells that express senescence markers increase in number during aging in both the dermal and epidermal layers of the skin [6-9]. Mitochondrial dysfunction is known to be both a cause and a consequence of cellular senescence in cultured cells [10-17]. Mitochondria continuously generate potentially damaging reactive oxygen species (ROS) during oxidative phosphorylation [18,19]. These ROS are continuously detoxified by cellular antioxidants and antioxidant defense enzymes. However, when ROS are excessive the resulting cellular damage can drive cellular senescence [20]. Despite numerous studies on mitochondrial function and senescence in cultured cells, little is known about the contribution of mitochondrial oxidative damage to cellular senescence *in vivo*.

Superoxide dismutase 2 (SOD2), also termed manganese superoxide (MnSOD), is the main antioxidant enzyme that scavenges ROS (specifically superoxide) in the inner mitochondrial matrix, and acts as a first line of defense against mitochondrial oxidative damage [21]. Mice that constitutively lack the *Sod2* gene develop several severe pathologies associated with aging within days after birth [22-24]. Although *Sod2*-deficient (*Sod2*^−/−^) mice die soon after birth (~100% mortality by day 10) [23], treatment with chemical SOD/catalase mimetics can (EUK) prolong life for about 3 weeks and attenuates many of the oxidative stress-associated pathologies [25]. Notably, embryonic fibroblasts (MEFs) cultured from *Sod2*^−/−^ mice proliferate slowly and have many more chromosome breaks, end-to-end fusions, and translocations than wild type (WT) MEFs [26]. Thus, *Sod2*^−/−^ MEFs may senesce more readily than WT MEFs in culture, consistent with oxidative stress causing severe DNA damage and subsequent senescence in mouse and human cells in culture [27,28].

Oxidative stress is thought to be a pivotal mechanism leading to skin aging [29]. Skin functions as a protective barrier that is essential to life, but the barrier and wound-healing functions decline with age [30]. Because of its high proliferative capacity and susceptibility to carcinogenesis, the skin makes an ideal model of mitochondrially driven cellular senescence *in vivo*.

Here, we provide evidence that mitochondrial oxidative stress promotes cellular senescence of the skin *in vivo*. In naturally aged mice, we show a decrease in cytologically detectable complex II activity and an increase in the frequency of senescent cells. We observe similar phenotypes in *Sod2*^−/−^ mice at very young ages. In *Sod2*^−/−^ mice, there is also significant epidermal thinning, which is an age-associated phenotype in mice and humans. We further demonstrate that mitochondrial dysfunction caused by rotenone promotes cellular senescence in human epidermal keratinocytes in culture. Our findings support the idea that mitochondrial oxidative damage can drive cellular senescence and aging phenotypes in the skin *in vivo*.

## RESULTS

### Increased cellular senescence and impaired mito-chondrial activity in aged mouse skin

**Figure 1:**
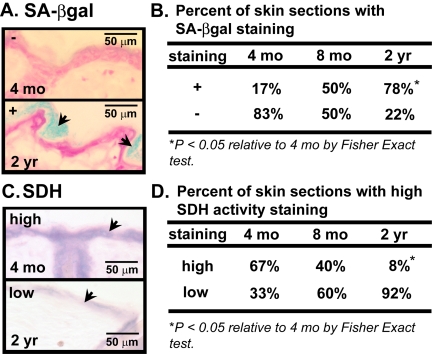
Cellular senescence and mitochondrial activity in skin of aging mice (**A**) Representative photomicrographs of dorsal skin sections from C57BL/6J WT mice, aged 4 mos or 2 yrs, stained for SA-βgal activity (blue) and counterstained with nuclear fast red (red). Arrows indicate SA-βgal+ cells. (**B**) Percent of C57BL/6J WT mice, aged 4 mos, 8 mos or 2 yrs, with SA-βgal positive (+) or negative (−) activity. (**C**) Representative photomicrographs of dorsal skin sections from the same mice, aged 4 mos or 2 yrs, stained for succinate dehydrogenase (SDH) (blue) activity. Arrows indicate staining in epidermis. (**D**) Percent of the same mice, aged 4 mos, 8 mos or 2 yrs, with SDH positive (+) or negative (−) activity. A total of six 4-month old (3 males and 3 females), ten 8-month old (5 males and 5 females), and thirteen 24-month old (7 males and 6 females) mice were analyzed.

We have previously shown an age-dependent increase in the number of senescent cells in human skin [6]. To verify that there is a similar increase in mouse skin, we used senescence-associated beta-galactosidase (SA-βgal) activity to identify senescent cells in the skin of young (4 mos old), middle-aged (8 mos old), and old (24 mos old) mice. Senescent cells stain blue when incubated with the substrate X-gal (pH 6.0) [6] due to increased expression of an endogenous beta-galactosidase activity [31]. SA-βgal activity (+) increased significantly in several areas of the stratum corneum (outermost layer of the epidermis, also referred as the cornified layer) as a function of age. A majority (78%) of skin sections from old mice had SA-βgal activity staining (+), while only 50% of skin sections from middle-aged mice and 17% of skin sections from young mice showed SA-βgal positivity (Figure 1A and B).

Because mitochondrial dysfunction is implicated as both a cause and consequence of senescence [10-17] we stained skin sections from young, middle-aged and old mice for succinate dehydrogenase (SDH), a measure of mitochondrial electron transport chain complex II activity. The proportion of sections with high SDH activity declined with increasing age of the mice. Whereas most (67%) sections from young mice showed high SDH activity in the epidermis, only 40% of sections from middle-age mice and 8% of sections from old mice had high SDH activity (Figure 1C and D). Although these could reflect age-dependent differences in chromogenic substrate accessibility or a decline in total mitochondrial copy number, the data suggests that aging entails both impaired mitochondrial activity and increased cellular senescence in the skin.

### Sod2 deficiency impairs mitochondrial activity and induces DNA damage

Elevated intracellular ROS is thought to both establish the senescent state and drive a positive feedback loop to maintain senescence [32, 33]. The nuclearly encoded and mitochondrially localized protein SOD2 reduces mitochondrial superoxide levels, and hence, decreases mitochondrial oxidative damage [21]. We previously showed that *Sod2*^−/−^ MEFs had higher superoxide levels than WT MEFs [26]., and *Sod2*^−/−^ mice had impaired mitochondrial activity in brain, heart, liver, and skeletal muscle tissues [23, 34]. To determine the relationship between mitochondrial dysfunction and cellular senescence in the skin, we used *Sod2*^−/−^ mice as a model. These mice lack a functional *Sod2* gene owing to a recombinational insertion that deletes exon 3 [23].

**Figure 2:**
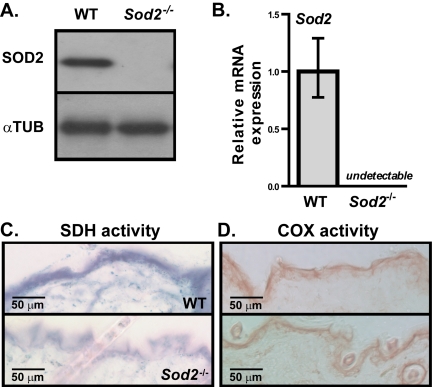
Sod2 expression and mitochondrial activity in skin of WT and *Sod2*^−/−^ mice (**A**) Western analysis for SOD2 and α-tubulin (αTUB) in dorsal skin samples from WT (n=6) and *Sod2*^−/−^ (n=6) mice, aged 17-20 days. (**B**) Quantitative PCR analysis of *Sod2* mRNA levels in skin of WT (n=8) and *Sod2*^−/−^ (n=9) mice. Transcript levels were normalized to beta-actin levels. Means with asterisks indicate significant differences at p<0.05 by Student's t test. (**C, D**) Representative photo-micrographs of skin sections from WT (n=8) and *Sod2*^−/−^ (n=9) mice, aged 17-20 days, stained for succinate dehydrogenase (SDH) (**C**; blue) and cytochrome c oxidase (COX) (**D**; brown) activities.

SOD2 protein is normally expressed in mouse skin. Using 17-20 days old, EUK-maintained WT and *Sod2*^−/−^ mice, we confirmed the complete absence of SOD2 protein and *Sod2* mRNA in the skin of *Sod2*^−/−^ but not WT mice by Western and qPCR analyses, respectively (Figure 2A and B). All *Sod2*^−/−^ mice examined showed decreased SDH activity in the epidermis and hair follicles relative to that of WT mice (Figure 2C), suggesting that *Sod2* deficiency impairs mitochondrial electron transport chain complex II activity. Interestingly, the epidermis of *Sod2*^−/−^ and WT mice had similar cytochrome c (COX) activity (Figure 2D), indicative of mitochondrial electron transport chain complex IV activity, consistent with our previous studies of other *Sod2*^−/−^ tissues [23, 34]. Complex II is composed of nuclear encoded proteins, while complex IV contains several mitochondrially encoded proteins.

**Figure 3:**
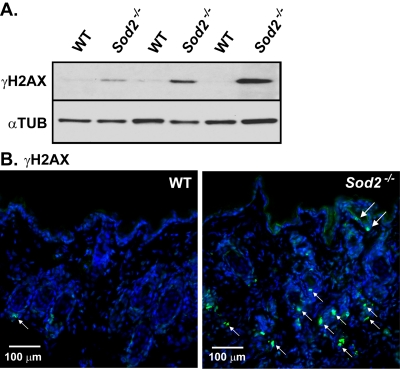
DNA damage in WT and *Sod2*^−/−^ mouse skin (**A**) Western analysis for γH2AX and α-tubulin (αTUB) in dorsal skin samples from WT (n=6) and *Sod2*^−/−^ (n=6) mice, aged 17-20 days. (**B**) Representative photomicrographs of immunofluorescence staining for γH2AX (green, arrows) staining of skin sections from WT (n=8) and *Sod2*^−/−^ (n=9) mice. Sections were counterstained with DAPI to identify nuclei (blue). Large arrows indicate staining in the epidermis; small arrows indicate staining in hair follicles.

ROS can also damage DNA [35], and DNA damage response (DDR) signaling is an important initiator and sustainer of the senescent state [1, 36-43]. To determine whether *Sod2* deficiency induces DNA damage in the skin, we scored cells for nuclear DNA double-strand breaks using the well-established marker phospho-rylated histone H2AX (γH2AX) [44,45]. Western analysis showed that γH2AX was essentially undetectable in skin from WT mice. By contrast, skin from *Sod2*^−/−^ mice had clearly detectable γH2AX levels (Figure 3A). In addition γH2AX-positive nuclei were rare in WT, but relatively abundant in *Sod2*^−/−^ epidermis and hair follicles by immunofluorescence (Figure 3B). These data suggest that *Sod2* deficiency can induce DNA double-strand breaks in the skin *in vivo*.

### Sod2 deficiency and mitochondrial oxidative damage promotes cellular senescence

**Figure 4:**
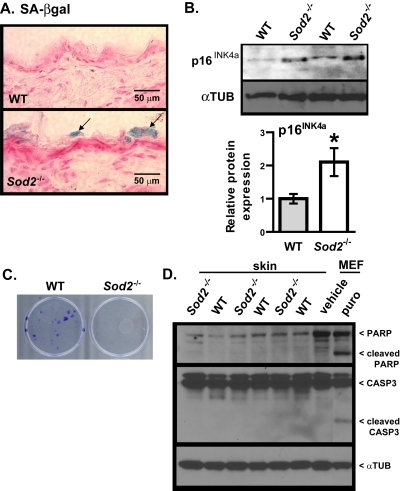
Cellular senescence in skin of WT and *Sod2*^−/−^ mice (**A**) Representative photomicrographs of skin sections from WT (n=8) and *Sod2*^−/−^ (n=9) mice, aged 17-20 days, stained for SA-gal activity (blue) and counter stained with nuclear fast red (red) to identify nuclei. (**B**) Western analysis for p16^INK4a^ and α-tubulin (αTUB) in dorsal skin samples from WT (n=6) and *Sod2*^−/−^ (n=6) mice. Bar graph (means ± SEM) is the average fold-change values of p16 protein normalized to αTUB. Means with asterisks indicate significant differences at p<0.05 by Student's t test. (**C**) Keratino-cytes were isolated from the skin of WT (n=3) and *Sod2*^−/−^ (n=3) mice, plated as described in Methods, and stained with crystal violet 20 d later. (**D**) Western analysis for intact and cleaved PARP and CASP3 and α-tubulin (αTUB) in dorsal skin samples from WT (n=6) and *Sod2*^−/−^ (n=6) mice. Also shown is a positive control of MEFs treated with vehicle (water) or 1μg/ml of puromycin (puro) for 4 days.

To determine whether the mitochondrial dysfunction and DNA damage in *Sod2*^−/−^ skin resulted in cellular senescence, we assayed WT and *Sod2*^−/−^ skin for SA-βgal activity. SA-βgal activity was detectable in several areas of the stratum corneum of all *Sod2*^−/−^ mice examined (n=9), but only minimal (only in 1 of 8 animals) activity was detected in the stratum corneum of WT mice (Figure 4A). Interestingly, no SA-βgal activity staining was observed in age-matched heterozygous (*Sod2*^+/−^) mice, which appear to have a gross skin phenotype similar to WT mice (not shown).

The p16^INK4a^ tumor suppressor protein is a key mediator of cellular senescence [1, 2, 46] and also a robust biomarker of aging in mice and humans, including skin [ii,^47^]. We therefore measured p16^INK4a^ expression in WT and *Sod2*^−/−^ mouse skin by Western analysis. p16^INK4a^ protein levels were two-fold higher in *Sod2*^−/−^ relative to WT skin (Figure 4B).

Consistent with increased cellular senescence in *Sod2*^−/−^ mouse skin, keratinocytes isolated from dorsal skin of these mice failed to form colonies in culture, in sharp contrast to keratinocytes isolated from WT mice (Figure 4C). Because apoptosis can also prevent the clonal expansion of cells, we measured the extent of poly-ADP-ribose polymerase (PARP) and caspase-3 (CASP3) cleavage, markers of apoptosis. WT and *Sod2*^−/−^ skin had similar levels of full length PARP and CASP-3, with little or no evidence of cleavage (Figure 4D). As a positive control, we treated WT MEFs with the pro-apoptotic drug puromycin. This showed increased PARP and CASP3 cleavage in these but not untreated MEFs (Figure 4D). These results suggest that *Sod2* deficiency in mouse skin promotes cellular senescence but not apoptosis.

Mitochondrial oxidative damage also promotes senescence in human cells. As confirmation, we treated human fibroblasts (HCA2) and keratinocytes (AG21837) in culture with low doses of rotenone, which inhibits mitochondrial complex I activity, resulting in increased ROS production and oxidative damage [48]. Rotenone inhibited cell proliferation, as indicated by fewer population doublings (PD) over 4 days (Supplementary figure 1A), and increased percentage of cells expressing SA-βgal (Supplementary figure 1B), consistent with previously reported results [49]. Interestingly, keratinocytes were more sensitive to rotenone-induced senescence than fibroblasts (Supplementary Figure 1B).

### Epidermal thinning in Sod2^−/−^ mice

**Figure 5:**
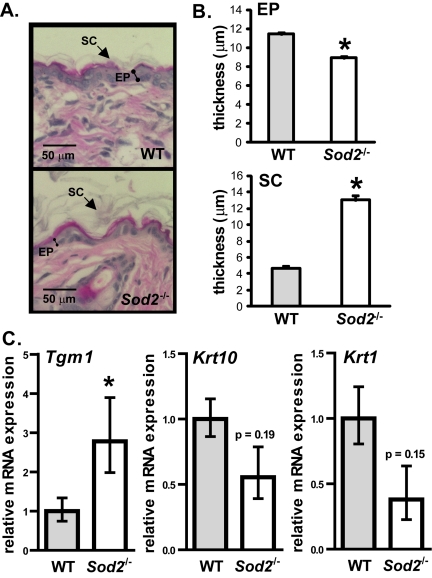
Epidermal thinning in *Sod2*^−/−^ mice (**A**) Representative photomicrographs of H&E staining of dorsal skin sections from WT (n=8) and *Sod2*^−/−^ (n=9) mice, aged 17-20 days. (**B**) Quantitation (means ± SEM) of the thickness of the epidermis (EP) and stratum corneum (SC) in skin sections from WT (n=8) and *Sod2*^−/−^ (n=9) mice using Image J software. (**C**) Quantitative PCR analysis of transglutaminase 1 (*Tgm1*), keratin 10 (*Krt10*) and keratin 1 (*Krt1*) mRNA levels in dorsal skin of WT (n=8) and *Sod2*^−/−^ (n=9) mice, aged 17-20 days. Transcript levels were normalized to beta-actin levels. Means with asterisks indicate significant differences at p<0.05 by Student's t test.

To determine the effect of *Sod2* deficiency on skin phenotype, we analyzed the histology of WT and *Sod2*^−/−^ skin. *Sod2*^−/−^ mice had a thinner epidermis (containing nucleated cells) relative to WT mice (Figure 5A and B). This thinning appeared to be due to fewer cells in the epidermis (Figure 5A), consistent with less proliferation due to senescence. Interestingly, *Sod2*^−/−^ skin also had a thicker stratum corneum (Figure 5A and B). This layer is composed of terminally differentiated anucleated cells that originate from the basal layer of the epidermis [50].

To test the idea that *Sod2* deficiency alters epidermal differentiation, we measured mRNA levels of transglutaminase 1 (*Tgm1*), a marker of terminally differentiating keratinocytes [51]. *Tgm1* mRNA levels were significantly (3-fold) higher in *Sod2*^−/−^ compared to WT skin (Figure 5C), suggesting accelerated terminal differentiation in the epidermis of these mice. By contrast, mRNA levels of keratins 1 (*Krt1*) and 10 (*Krt10*), markers of transit amplifying cells and early differentiated cells in the epidermis [52,53], were somewhat lower in *Sod2*^−/−^ skin, although this difference was not statistically significant (Figure 5C). These data suggest that *Sod2* deficiency promotes terminal differentiation in keratinocytes, resulting in excessive cornified layer production. Increased terminal differentiation and decreased proliferation due to senescent cells may collectively contribute to the decreased epidermal thickness in *Sod2*^−/−^ mice.

## DISCUSSION

Here, we show how natural aging is accompanied by decreased mitochondrial activity and increased cellular senescence in mouse skin. Consistent with the idea that these events are causally linked, constitutive *Sod2* deficiency resulted in mitochondrial dysfunction and cellular senescence in the epidermis, as well as epidermal thinning, a known feature of aging skin.

The presence of senescent cells in *Sod2*-deficient skin is associated with increased nuclear DNA damage, a potent inducer of senescence [1, 36-43]. This damage could result from the increase in ROS that occurs in the absence of SOD2. Alternatively, it could result from reduced DNA repair as a consequence of diminished ATP production, or a decrease in the synthesis of heme, which is needed for enzymes that participate in DNA replication and repair. In any case, our data provides evidence that mitochondrial oxidative damage can cause cellular senescence in the skin *in vivo*.

A recently developed mouse model of conditional *Sod2* deficiency in connective tissue [54] had a reduced lifespan, accelerated aging phenotypes (e.g., weight loss, skin atrophy, kyphosis, osteoporosis, muscle degeneration) and increased cellular senescence by postnatal day 150. We found that constitutive *Sod2* deficiency accelerated aging phenotypes and cellular senescence in the skin at postnatal days 19-20. The early appearance of senescent cells in the epidermis of constitutive *Sod2*-deficient mice, as opposed to their later appearance in connective tissues of conditional *Sod2*-deficient mice might be attributed to the location of epidermal cells at the interface between the body and environment. The epidermis is directly exposed to a highly pro-oxidative environment and may be especially vulnerable to oxidative stress [55]. Interestingly, human keratinocytes were more susceptible to rotenone-induced senescence than human skin fibroblasts. Keratinocytes are thought to preferentially use their mitochondria for ATP production, even at the expense of superoxide production [56]. Moreover, unlike fibroblasts, which proliferate better in 3% oxygen [27], keratinocytes proliferate better in 20% oxygen [57]. Thus, mitochondrial oxidative damage caused by *Sod2* loss may be more significant in keratinocytes than fibroblasts, consistent with senescent cells localizing primarily in the epidermis of *Sod2*^−/−^ mice.

The epidermis tends to thin with increasing age in humans and mice [58,59]. The presence of senescent cells in the aging mouse epidermis may partially explain epidermal thinning in aging skin. We speculate that these senescent cells arise from rapidly proliferating transit-amplifying cells, as the proliferation kinetics of such cells are thought to influence epidermal aging more so than stem cells [60]. Cell proliferation in the epidermis is due predominantly to transit-amplifying cells, which reside in the suprabasal layer [53]. A defect in the proliferation of transit-amplifying cells could lead to epidermal hypoplasia and epidermal thinning. Consistent with this view, *Sod2*^−/−^ skin trended towards lower expression of *Krt1* and *Krt10*, markers of transit amplifying cells, and we previously de-monstrated that senescent cells increase with age in the suprabasal layer [6].

Senescent keratinocytes can differentiate in culture, albeit with an aberrant gene expression profile [61]. We found SA-βgal activity primarily in the terminally differentiated stratum corneum, supporting the idea that senescent keratinocytes remain capable of differentiation. Because SA-Bgal expression is a relatively late senescence event [62], the rapid differentiation of mouse keratinocytes (~3-4 days in culture) [63], may have allowed enough time for the senescent cells to differentiate to the outer layer of the epidermis. Further, mitochondria undergo dramatic remodeling during the differentiation of keratinocytes, which is necessary for the differentiation [64], and dysfunctional mitochodria can accelerate keratinocyte differentiation without inducing apoptosis (^v^). Our finding that *Sod2*^−/−^ skin expresses elevated levels of *Tgm1*, a marker of terminal keratinocyte differentiation is consistent with the increased thickness of *Sod2*^−/−^ stratum corneum reflecting increased keratinocyte differentiation.

In summary, we provide *in vivo* evidence for a causal relationship between mitochondrial oxidative damage, cellular senescence and aging phenotypes in the skin. Our findings open avenues for understanding how mitochondrial dysfunction and senescent cells influence aging phenotypes in the skin.

## METHODS

### Cell culture

Human keratinocytes (AG21837) were obtained from the Coriell Cell Repository (Camden, NJ, USA) and cultured in keratinocyte growth medium (CnT-07 medium, Zenbio, Research Triangle Park, NC, USA) with penicillin-streptomycin (Invitrogen, Carlsbad, CA, USA) in 20% oxygen. Human HCA2 fibroblasts were cultured in DMEM (Invitrogen) with 10% FBS and penicillin-streptomycin in 3% oxygen. Experiments were performed in 20% oxygen. Media were replaced every 2–3 d. MEFs were cultured at 3% oxygen in DMEM (Invitrogen) with 10% FBS and penicillin-streptomycin.

### Population doubling

Cells were seeded at 5 × 10^4^ cells/well in six well plates and treated with vehicle (DMSO) or rotenone (Sigma-Aldrich, St. Louis, MO, USA) for 4 d. Media were refreshed every 2 d. Cells were trypsinized and counted using a Beckman Coulter counter.

### Animal experiments

Animal studies were conducted in compliance with protocols approved by our Institutional Animal Care and Use Committee. CD1 WT and *Sod2*^−/−^ mice were bred as previously described [23]. Offsprings were generated from heterozygous matings. Because *Sod2*^−/−^ mice die soon after birth, all mice were treated with 1mg/kg of EUK-189 per day starting at postnatal day 3, as described [25]. Skin samples from 17-20 day old postnatal mice were collected. For aging studies, C57BL/6J mice were purchased from the Jackson Laboratory. Males and females were combined according to age to increase statistical power.

### Clonogenicity assay

Keratinocytes were isolated and cultured as described with some modification [66]. Briefly, shaved dorsal skin of 17-20 d old WT and *Sod2*^−/−^ mice were dissected, washed with PBS, Hibiclens (Fisher Scientific), and calcium/magnesium-free HBSS (HBSS-cmf, Invitrogen) containing 5x penicillin-streptomycin, and floated [dermis side down] on 1 ml dispase (25 U/mL; BD Biosciences, Bedford, MA, USA) and 0.05 mg/mL of gentamicin (Invitrogen) in HBSS-cmf] at 4°C overnight. The epidermis was scraped from the dermis and incubated with TrypLE (Invitrogen) for 8 min at 37°C. TrypLE was neutralized with HBSS containing 1% chelexed-FBS (BioRad). After filtering through a 70 μm cell strainer, cells were seeded at 5 × 10^4^ cells in 35-mm plates and cultured in keratinocyte-specific growth medium (refreshed every 2 d). After 20 days, cells were fixed in 10% buffered formalin for 20 min and stained with crystal violet (0.5% crystal violet, 20% methanol in 1x PBS). Stained cells were scanned with a hpscanjet 4700c.

### Histology

Skin samples were fixed in 10% buffered formalin, processed for paraffin-embedding, cut into 4 μm sections, and stained with hematoxylin and eosin (H&E). Epidermal and stratum corneum thicknesses were measured using Image J software.

### Mitochondrial enzyme activity staining

Skin samples were embedded in frozen OCT medium, cut into 20 μm sections, and incubated with succinate dehydrogenase (SDH) and cyclooxygenase (COX) activity staining solution as described [23].

### Immunohistochemistry

OCT-embedded samples were cut into 10 μm sections. Sections were fixed in 10% buffered formalin, permeabilized with 0.5% triton-X, blocked with 4% donkey serum/1% BSA in PBS solution and incubated with anti-γH2AX (NB100-79967, Novus Biologicals, 1:500) overnight at 4°C, followed by incubi-tion with Alexa 555 donkey anti-rabbit (Invitrogen, 1:750) for 1 h at room temperature. Sections were mounted with Prolong Gold with DAPI (Invitrogen).

### SA-βgal activity staining

Cells were processed for SA-βgal staining using Senescence Detection Kit (BioVision, Mountain View, CA, USA) and bright field and phase contrast microscopy. Percent SA-βgal positivity was computed as the number of blue cells over the number of total cells. For skin, tissues were cut into 10 μm sections and processed using the same kit. Tissues were counterstained with nuclear fast red and visualized by brightfield microscopy. Four fields were taken at 40X magnification for each skin section and three sections per animal were analyzed. Skin samples with several SA-βgal staining (blue staining in at least two out of four fields) were considered as SA-βgal positive for that tissue.

### RNA isolation and analysis

Samples were lysed by QIAzol Lysis Reagent following the manufacture's protocol (Qiagen, Valencia, CA, USA). RNA was isolated using an RNeasy Tissue Mini Kit with DNase treatment and QIAcube system (Qiagen). RNA was quantified, and integrity assessed by Agilent Bioanalyzer 2100 (Agilent Technologies, Inc., Santa Clara, CA, USA). cDNAs were synthesized using random primers and iScript RT reagents following the manufacturer's protocol (Bio-Rad Laboratories, Hercules, CA, USA) and quantified by real-time quantitative PCR using the Roche Universal Probe Library system (Indianapolis, IN, USA). The primer sets (0.1 μM) were as follows: 1) *Sod2*: 5'-CCA TTT TCT GGA CAA ACC TGA-3' and 5'- GAC CCA AAG TCA CGC TTG ATA-3' with probe #67, 2) *Tgm1*: 5'-GCC CTT GAG CTC CTC ATT G-3' and 5'-CCC TTA CCC ACT GGG ATG AT-3' with probe #10, 3) *Krt1*: 5'-TTT GCC TCC TTC ATC GAC A-3' and 5'-GTT TTG GGT CCG GGT TGT-3' with probe #62, 4) *Krt10*: 5'- CGT ACT GTT CAG GGT CTG GAG-3' and 5'-GCT TCC AGC GAT TGT TTC A-3' with probe #95, 5) Actb: 5'-CTA AGG CCA ACC GTG AAA AG-3' and 5'-ACC AGA GGC ATA CAG GGA CA-3' with probe #64, and 6) 16S: 5'-AAA CAG CTT TTA ACC ATT GTA GGC-3' and 5'-TTG AGC TTG AAC GCT TTC TTT A-3' with probe #83. cDNAs (1 μg RNA) were amplified by TaqMan Universal PCR Master Mix (Applied Biosystems) as follows: 95°C for 10 min and 40 cycles of 95°C for 15 sec, 70°C for 5 sec, and 60°C for 1min. Transcript levels were normalized to beta-actin (Actb) levels, which were compared to 16S RNA levels to validate Actb as a normalization gene.

### Protein isolation and Western analysis

Proteins were isolated using RIPA lysis solution (Santa Cruz Biotechnologies, Santa Cruz, CA, USA), separated by electrophoresis on 4-12% polyacrylamide gels (NuPAGE Bis-Tris Gel, Invitrogen), and transferred to PVDF membranes. Membranes were blocked with 5% milk in TBS-T and incubated with primary antibodies as follows: anti-γH2AX (NB100-79967, Novus Biologicals, 1:1500), αTUB (T5168, Sigma Aldrich, 1:4000), PARP (9542, Cell Signaling Technologies, 1:1000), CASP3 (9665, Cell Signaling Technologies, 1:1000), p16 (sc-1207, Santa Cruz, 1:500). Membranes were incubated with appropriate HRP-conjugated secondary antibody against rabbit (1:1000) or mouse (1:2000) IgG (BioRad).

### Data analysis

Data are presented as the least square means ± SEM and were statistically analyzed using the Student's t test. P <0.05 was considered statistically significant. Fisher Exact tests were performed on SDH and SA-βgal activity staining.

## SUPPLEMENTARY FIGURE

Supplementary Figure 1 Cellular senescence in human keratinocytes and skin fibroblasts treated with rotenone (**A**) Proliferation (number of population doubling in 4 days) of primary human skin fibroblasts (HCA2, green) and keratinocytes (AG21837, blue) treated with various doses of rotenone for 4 days. (**B**) Quantitation of the percentage of HCA2 and AG21837 cells with positive SA-βgal staining after treatment with 100 nM rotenone for 9 days. Bar graphs are presented as least square means ± SEM. Means with asterisks indicate significant differences at p<0.05 by Student's t test.
All measurements were done in quadruplicates.
