# Supplementary figures and images for: Mitochondrial oxidative stress caused by Sod2 deficiency promotes cellular senescence and aging phenotypes in the skin
Source: Aging (Albany NY). 2012 Jan 20;4(1):3–12. doi: 10.18632/aging.100423 (PMC3292901; doi:10.18632/aging.100423)

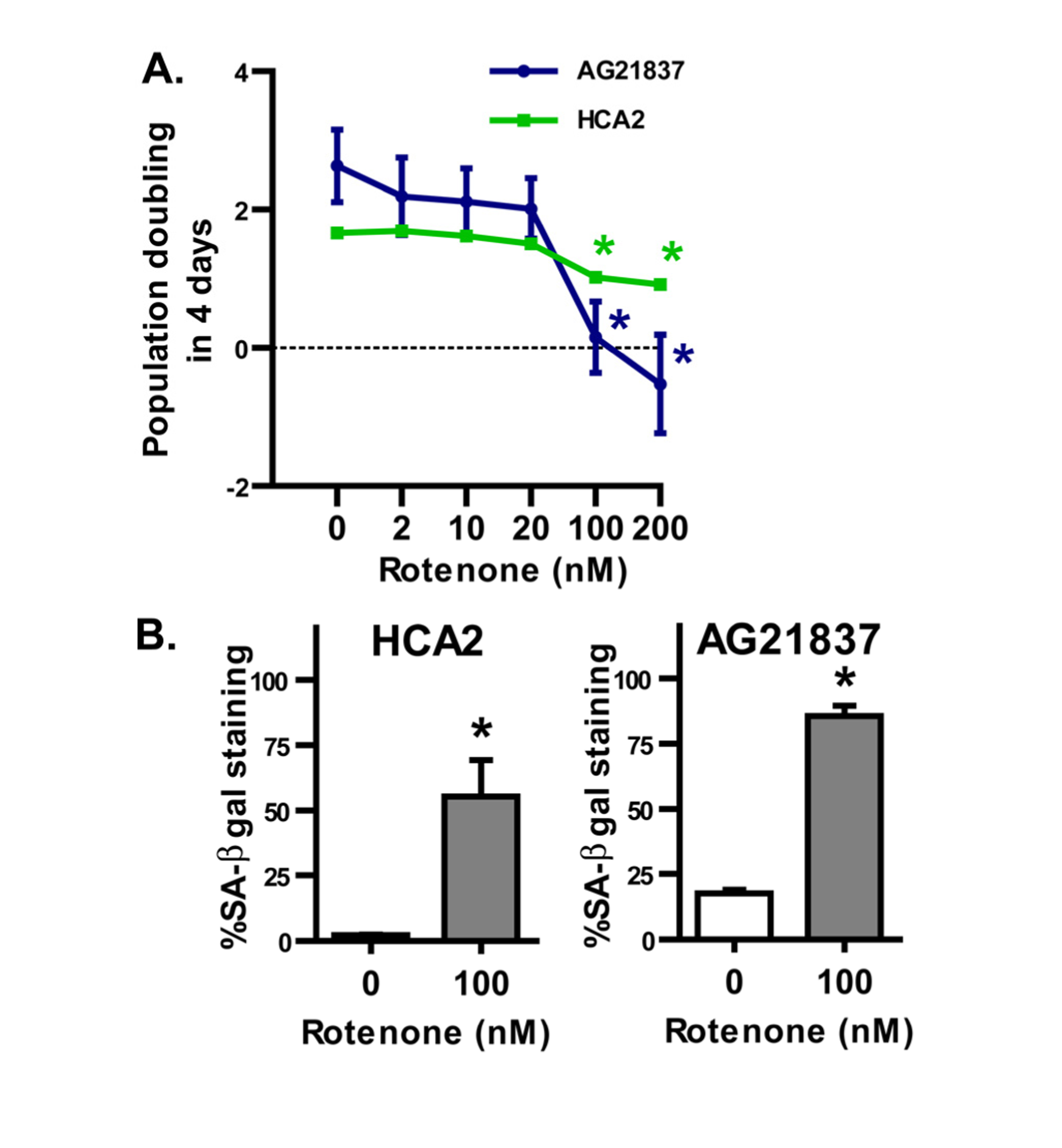

Supplement: Supplementary Figure 1 — (A) Proliferation (number of population doubling in 4 days) of primary human skin fibroblasts (HCA2, green) and keratinocytes (AG21837, blue) treated with various doses of rotenone for 4 days. (B) Quantitation of the percentage of HCA2 and AG21837 cells with positive SA-βgal staining after treatment with 100 nM rotenone for 9 days. Bar graphs are presented as least square means ± SEM. Means with asterisks indicate significant differences at p<0.05 by Student's t test. All measurements were done in quadruplicates. [file aging-04-003-s001.tif]
